# Predictors of maternal psychological distress in rural India: A cross-sectional community-based study

**DOI:** 10.1016/j.jad.2012.01.029

**Published:** 2012-05

**Authors:** Audrey Prost, Rashmi Lakshminarayana, Nirmala Nair, Prasanta Tripathy, Andrew Copas, Rajendra Mahapatra, Shibanand Rath, Raj Kumar Gope, Suchitra Rath, Aparna Bajpai, Vikram Patel, Anthony Costello

**Affiliations:** a UCL Centre for International Health and Development, Institute of Child Health, University College London, UK; b Effective Intervention, Centre for Economic Performance, London School of Economics and Political Science, London, UK; c Ekjut, Chakradharpur, Jharkhand, India; d UCL Centre for Sexual Health & HIV Research & MRC Clinical Trials Unit, UK; e London School of Hygiene and Tropical Medicine, Keppel Street, London WC1E 7HT, UK; f Sangath, Goa, India

**Keywords:** Common mental disorder, Maternal depression, India, Rural health

## Abstract

**Background:**

Maternal common mental disorders are prevalent in low-resource settings and have far-reaching consequences for maternal and child health. We assessed the prevalence and predictors of psychological distress as a proxy for common mental disorders among mothers in rural Jharkhand and Orissa, eastern India, where over 40% of the population live below the poverty line and access to reproductive and mental health services is low.

**Method:**

We screened 5801 mothers around 6 weeks after delivery using the Kessler-10 item scale, and identified predictors of distress using multiple hierarchical logistic regression.

**Results:**

11.5% (95% CI: 10.7–12.3) of mothers had symptoms of distress (K10 score > 15). High maternal age, low asset ownership, health problems in the antepartum, delivery or postpartum periods, caesarean section, an unwanted pregnancy for the mother, small perceived infant size and a stillbirth or neonatal death were all independently associated with an increased risk of distress. The loss of an infant or an unwanted pregnancy increased the risk of distress considerably (AORs: 7.06 95% CI: 5.51–9.04 and 1.49, 95% CI: 1.12–1.97, respectively).

**Limitations:**

We did not collect data on antepartum depression, domestic violence or a mother's past birth history, and were therefore unable to examine the importance of these factors as predictors of psychological distress.

**Conclusions:**

Mothers living in underserved areas of India who experience infant loss, an unwanted pregnancy, health problems in the perinatal and postpartum periods and socio-economic disadvantage are at increased risk of distress and require access to reproductive healthcare with integrated mental health interventions.

## 1 Introduction

Maternal common mental disorders, characterised by significant levels of depressive, anxiety and somatic symptoms, are prevalent in low and middle-income countries: between 15 and 57% of women screen positive for symptoms of depression during pregnancy or the postpartum period (Engle, 2009; Wachs et al., 2009). The adverse consequences of poor maternal mental health are far-reaching for both mothers and children. Mothers affected by common mental disorders may be less able to care for their own health or have reduced ability to mobilise social support during the perinatal period; in addition, studies have demonstrated that children born to depressed mothers are at increased risk of poor physical growth (Stewart, 2007; Surkan et al., 2011). South Asian studies have also shown that mothers with common mental disorders, in particular depression, are more likely to have infants who are of low birth weight, and become underweight and stunted in early childhood (Black et al., 2009; Gausia et al., 2009; Patel and Prince, 2006; Patel et al., 2004). Commonly identified predictors of maternal common mental disorders in South Asia include low socio-economic status, lack of social support, adverse life events, disappointment with the sex of the baby and a bad relationship with a mother-in-law or partner (Chandran et al., 2002). In India, estimates of maternal depression among women accessing antenatal care range from 11.9 to 23% (Chandran et al., 2002; Patel et al., 2003). Women from the poorest social groups are likely to be the worst affected (Patel et al., 2002). Scheduled Tribes or *adivasis* (‘indigenous inhabitants’) represent 84.3 million people or 8.2% of India's population, are among the country's poorest communities, and have higher morbidity and mortality rates compared with non-indigenous populations (Government of India, 2011; Subramanian et al., 2006). In this study we aimed to identify socio-economic, gender and health-related predictors of maternal psychological distress, a proxy for maternal common mental disorders, among recently delivered mothers within a community-based sample in rural, tribal areas of eastern India.

## 2 Methods

### 2.1 Setting

In an earlier study we reported data from a cluster-randomised controlled trial testing the impact of a participatory intervention with women's groups on neonatal mortality and maternal psychological distress in three districts of Jharkhand and Orissa, in eastern India (Tripathy et al., 2010). In this study we use data from the trial's 18 control clusters to understand predictors of maternal psychological distress. Data on distress were collected over 24 months in the second and third years of the trial (2006–2008). We only use data from the trial's control clusters because the trial intervention – a participatory learning and action cycle with women's groups – led to a 57% reduction in postpartum psychological distress in the third year of the study (OR: 0.43, 95% CI: 0.23–0.80), and the intervention is likely to have affected predictors of distress such as neonatal mortality, health problems and lack of social support in the perinatal period. The 18 geographic control clusters had a mean population of 6341 (range: 3589–7453), covering an estimated overall population of 114,141 in three rural districts of Jharkhand and Orissa. Over 60% of mothers were from communities often referred to as Scheduled Tribes or *adivasi* (meaning ‘original inhabitant’), including Ho, Santhal, Bhumij, Juang, Bhuiyan and Munda groups. The study area also included groups from Scheduled Castes (SC) and Other Backward Classes (OBC). ST, SC and OBC are legal designations to identify socially and economically disadvantaged groups in order to facilitate affirmative action in access to education, government employment, and political office. However these labels are regarded by many as simplistic political constructs masking a more complex social reality (Dirks, 2001; Guha, 1999): SCs, STs and OBCs living in the same area may have similar social practices and livelihoods and there is heterogeneity in economic status within these groups (Corbridge, 2000; Guru and Chakravarty, 2005). A common characteristic of many groups within our study clusters, however, was geographical isolation: only 52% of (97/185) villages in the study areas had a Primary Health Centre within 10 km (Rath et al., 2010). Although some of the study districts have a District Mental Health Programme and at least one NGO is doing mental health work in Jharkhand (Kumari et al., 2009), the coverage of mental health services remains sporadic and insufficient.

Child and maternal mortality rates in the study area were high: during the trial baseline the neonatal mortality rate (NMR) was 58 per 1000 live births and the maternal mortality ratio was 510 per 100,000 live births. While neonatal mortality decreased substantially in the trial's intervention clusters as a result of the women's group meetings, it remained high (> 50 per 1000 live births), in the control clusters from which data for this study are drawn. Only 40% of women in control clusters had three or more antenatal care visits and 80% delivered their children at home (Tripathy et al., 2010).

### 2.2 Participants and assessment

Data were available for 5801 mothers who gave birth between July 31st, 2006, and July 30th, 2008 in 18 population clusters. Participants (mothers) were identified as follows: community-based key informants were responsible for reporting any births, and maternal and neonatal deaths within a catchment area of 250 households. Interviewers verified births and deaths and all consenting mothers were given a structured questionnaire interview around 6 weeks after delivery (Barnett et al., 2008). Follow-up and response rates were high (> 90%) and are described in detail elsewhere (Tripathy et al., 2010). The questionnaire captured information on the mother's socio-demographic background and events during the antepartum, delivery and postpartum periods. After examining the existing literature on maternal common mental disorders including depression, we selected the following potential predictors for analysis:

(a) *Socio-economic factors* including maternal age; education; literacy; caste or tribal group; land and asset ownership; possession of a Below Poverty Line card which entitles one's household to subsidized food grain, healthcare and housing.
(b) *Gender-related factors* including mother's decision-making power concerning daily household expenditure and healthcare-seeking for herself in case of illness; food restrictions in the postpartum period (only one meal a day).
(c) *Physical and reproductive health factors* including parity, caesarean section, consumption of alcohol in pregnancy, and unwanted pregnancy by the mother or father
  Health problems during pregnancy, including at least one of the following: severe stomach pain; excessive vomiting; fever for more than 24 h; excessive vaginal bleeding; jaundice; reduced/no foetal movement; self-reported symptoms of malaria.
  Health problems during delivery, including at least one of the following: high fever in the 3 days before labour, foul smelling vaginal discharge; prolonged labour; fits or convulsions; vaginal bleeding; retained placenta; tear around birth passage; umbilical cord around infant's neck.
  Health problems in the postpartum period (the first 6 weeks after birth), including at least one of the following: severe stomach pain; fever for more than 24 h; foul smelling discharge; excessive vaginal bleeding; leaking from vagina.
(d) *Factors related to the birth outcome*, including mother perceiving her infant to be smaller than average; stillbirth (infant never cried or breathed after delivery) and neonatal death (death between birth and 28 completed days of life), grouped into one variable (infant loss).

We did not ask about previous history of miscarriages, stillbirths, adverse life events, or domestic violence. The K10, a screening tool for non-specific psychological distress, was introduced in the study questionnaire following training by a psychiatrist [RL] (Kessler et al., 2002). It was selected in preference to a clinical diagnostic interview because of its brevity and ease of use by lay interviewers in large surveys, and due to its reliability in identifying common mental disorders as compared to four other screening questionnaires (Patel et al., 2008). The tool has been validated to screen for common mental disorders in developing country settings (Kessler and Üstün, 2008) and has previously been used in India (Patel et al., 2008). It consists of ten questions to elicit the frequency of depressive and anxiety symptoms over the past month on a 4-point Likert scale of frequency. This is used to generate a score (range: 0–50) measuring the severity of common mental disorder symptoms. We followed recommendations from previous studies that suggested a cut-off between 5 and 6, which corresponds to a score of 15 or more, as acceptable against the ICD-10 diagnosis for any common mental disorder criterion (Andrews and Slade, 2001; Patel et al., 2008).

#### 2.2.1 Ethical considerations

The study from which our data are drawn was granted ethical approval by an independent ethical review committee in Jamshedpur, India, and by the Ethics Committee of the Institute of Child Health and Great Ormond Street Hospital. We made efforts to refer women with severe depression to primary care services or to the tertiary hospital in Ranchi, Jharkhand.

### 2.3 Data analyses

**Fig. 1:**
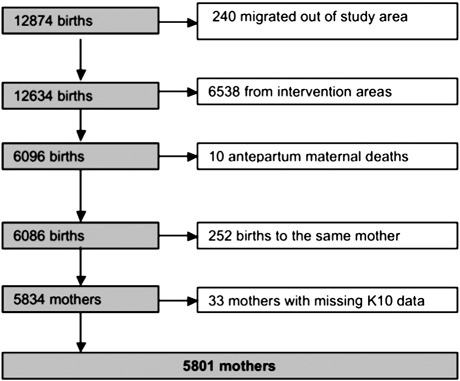
Flowchart describing the selection of cases for the study.

Data from 12,874 births were available from years 2 and 3 of the earlier cluster-randomised controlled trial, when data on psychological distress were collected. From these we excluded births to mothers who migrated outside the study areas and for whom details on psychological distress could not be obtained, as well as births from the trial intervention clusters, and maternal deaths. If mothers had delivered more than one child during the study period, either because of multiple births or through repeated pregnancies, we only included the first child of a multiple birth and the first child in the study period. Fig. 1 shows how participants were selected for analyses. Since over 60% of mothers had a K10 score of 10 we recoded scores into a categoric binary variable and categorised psychological distress as either low to mild (K10 score < 16) or moderate to severe (K10 = 16–40) following the cut-off points recommended by Andrews and Slade (2001) and Patel et al. (2008). We grouped moderate and severe distress together because of the low numbers of women with severe distress. The association of each factor with psychological distress (K10 score > 15) was assessed through univariable analysis using simple logistic regression with random effects at the cluster level. We assessed the odds of distress according to the presence or absence of each predictor and calculated odds ratios with 95% confidence intervals. We entered all variables that showed an association at p ≤ 0.1 in univariable analyses into a multivariable logistic regression model with cluster-level random effects. Retention of variables in final models was based on backward selection and likelihood ratio tests. All analyses were carried out using Stata/IC 10.0 (Stata Corp, 2008). We excluded factors that could be consequences of antepartum or postpartum psychological distress, including feeding problems and weakness or tiredness during pregnancy and the postpartum period. Our analyses showed a strong impact of neonatal infant loss on distress, as well as significant interactions between infant loss and potential predictors of distress. We therefore present the association of potential predictors with distress for all mothers, report the results of interaction tests, then show results of analyses separately for mothers with and without an infant loss.

## 3 Results

### 3.1 Participant characteristics

Table 1 describes the maternal socio-demographic characteristics of the 5801 mothers included in the study. The mean maternal age was 25.5 years (SD 5.3). Over two thirds (66.3%) had not been to school, 67.8% belonged to a Scheduled Tribe and 27.2% to Other Backward Castes. Forty-three percent of mothers belonged to a household holding a BPL card. The sex ratio for births in this population was 950: 51.3% of infants were male and 48.7% female. Twenty-eight percent of mothers were primiparous and 77.5% of them delivered at home (data not shown in table).

### 3.2 Prevalence and predictors of psychological distress

The overall prevalence of maternal psychological distress (K10 > 15) in this study was 11.5% (669/5801, 95% CI: 10.7–12.3). The intracluster correlation coefficient for distress in this population, calculated using a large one-way ANOVA, was 0.08 (95% CI: 0.03–0.13).

Table 2 reports the results of univariable and multivariable analyses exploring the association of potential socio-economic, gender and health predictors with psychological distress for all mothers (n = 5801). We treated maternal age as an a priori confounding variable and included it in all models. We found no association between literacy, belonging to an underprivileged social group (Scheduled Caste or Other Backward Caste), having a Below Poverty Line card, alcohol consumption in pregnancy, food restrictions in the postpartum period or infant's sex with maternal psychological distress. Higher age, low asset ownership, health problems in the antepartum, delivery or postpartum periods, caesarean section, unwanted pregnancy for the mother, as well as small perceived infant size and an infant loss were all significantly associated with distress at p ≤ 0.1 in univariable analyses. In multivariable analyses, higher age, health problems in the antepartum, delivery or postpartum periods, small perceived infant size and infant loss remained significantly associated with distress. The loss of an infant or an unwanted pregnancy increased the risk of distress considerably (AORs: 7.06 95% CI: 5.51–9.04 and 1.49, 95% CI: 1.12–1.97, respectively).

We found an inverse association between mothers' decision-making power and distress. However 50.6% of mothers who reported taking decisions on their own belonged to the poorest asset quartile, as opposed to only 29.2% in households where husbands and in-laws were the main decision-makers (data not shown in table). This suggests that our measure of decision-making may mainly reflect poverty and social isolation.

There were significant interactions (p < 0.1) between low maternal education, higher age, land ownership, asset ownership, health problems in the perinatal period and unwanted pregnancy with infant loss. For this reason we conducted stratified univariable and multivariable analyses for all predictors, separating mothers with an infant loss (n = 456) and those whose infants survived the first month of life (n = 5345). Table 3 shows the results of these analyses. Among mothers whose infants survived, the predictors of distress were the same as for the sample as a whole. Only health problems during delivery were associated with distress in the final multivariable model for mothers who experienced an infant loss.

## 4 Discussion

### 4.1 Prevalence and predictors of postpartum psychological distress: new and confirmed findings

Our study has identified predictors of maternal psychological distress in a rural, largely tribal community sample in eastern India. The prevalence of psychological distress (11.5%) – used here as a proxy for maternal common mental disorders – was lower than in other studies from South Asia, most of which used either the Edinburgh Postnatal Depression Scale or a clinical diagnostic interview. In Goa, India, a study of women attending antenatal care reported a prevalence of 23.4%, compared with 19.8% in a community-based cohort from Tamil Nadu and 28% in a community cohort study in Pakistan (Patel and Prince, 2006). We used a cut-off score of 15 for the K10 following published recommendations but did not validate this threshold in our study population. The lower prevalence found in our study could therefore be an artefact of the K10 or of this cut-off point, though the K10 and other screening scales showed reasonable sensitivity and specificity against each other, and our low cut-off point means that we were more likely to over-report cases than under-report them (Patel et al., 2008).

Over two thirds of mothers in our study were from tribal groups. Another possible explanation for the relatively low prevalence of distress found in this study could be that some social characteristics specific to these communities are protective against maternal psychological distress. Previous South Asian research has identified son preference and poor marital relationships as important predictors of distress (Chandran et al., 2002; Patel et al., 2002). Some studies have argued that tribal communities exhibit less son preference than non-tribal groups and have a more equitable gender division of labour. Mitra (2008) explored the status of women among Scheduled Tribes using Indian Census data and reported higher sex ratios and female work participation rates in Scheduled Tribes compared to Scheduled Castes and other communities. In our study however the sex ratio was only slightly higher (950) than the national average (940) (Government of India, 2011). We also have little evidence to support the hypothesis of increased decision-making power among tribal mothers: around one third of mothers reported taking decisions about daily expenditure and care-seeking jointly with their husbands, but over 50% also said that husbands or in-laws were the main decision-makers in their household. We should therefore be cautious not to generalise about the impact of gender norms within this heterogeneous set of tribal communities.

We found that higher age and low asset ownership were associated with an increased risk of psychological distress. This supports findings from a recent study in which India's National Family Health Survey data (NFHS-3, 2005–6) were used to identify factors associated with common mental disorders in rural married Indian women, and in which both age and socio-economic status were associated with common mental disorders (Shidaye and Patel, 2010). Our data also broadly concur with findings from a recent systematic review, which found that poverty-related measures including education, social class, socio-economic status and financial stress were strongly and consistently associated with CMDs (Lund et al., 2010). The absence of an independent association between tribal status and psychological distress in our multivariable analyses may be due to the fact that most of the study respondents were tribal.

Our study confirms the importance of reproductive health for maternal mental health: problematic pregnancies and deliveries and those that resulted in a stillbirth or neonatal death put women at increased risk of psychological distress. A recent study from Benin showed that women who suffered from severe obstetric complications (near-miss events) did not have an increased risk of maternal depression except when they had a perinatal death, and that the birth of a live baby mitigated the impact of near-miss events on postpartum distress (Fottrell et al., 2010). Increased preventive care during pregnancy, delivery and the postpartum period is therefore likely to impact positively on maternal mental health and subsequently on the health of infants and older children in the household. The study also highlights the role of unwanted pregnancies in contributing to poor maternal mental health independently of health problems in the perinatal period. This is consistent with the results of a recent systematic review highlighting the increased risk of postpartum depression among rural mothers in developing countries with two or more children under 7 or more than five children (Villegas et al., 2011). Given the high levels of maternal mortality, neonatal mortality and child undernutrition in the study districts, this provides additional evidence that scaling up access to family planning should be a priority intervention in such underserved areas, as recommended in a recent call for action to scale up reproductive health programmes in 250 high-burden Indian districts (IIPS and Macro International, 2007; Paul et al., 2011).

### 4.2 Preventing and treating maternal depression in underserved areas

How could maternal common mental disorders in pregnancy and the postpartum period be prevented and treated in areas where the provision of mental health services is scarce? There is increasing evidence from low and middle-income countries that treatment for common mental disorders can be delivered by lay or community health workers, as well as by community groups. A recent trial from Pakistan showed that cognitive behaviour therapy delivered by community health workers to depressed women in the third trimester of pregnancy reduced the incidence of major depression in the postpartum period, and such methods could be adapted for other community health workers in rural settings (Rahman et al., 2008). In a collaborative care model recently tested in Goa, India, lay health counsellors worked with primary care and mental health specialists to provide psychosocial interventions and antidepressants to patients with common mental disorders; patients with confirmed ICD10 common mental disorders in the intervention group were more likely to have recovered at 6 months than those in the control group (RR 1.22, 95% CI: 1.00–1.47), though the effect was mainly observed in government facilities and not in private facilities (Patel et al., 2010). In Uganda, group interpersonal psychotherapy delivered through community groups led to reductions in mean depression scores and major depression among group members, and these improvements persisted 6 months after treatment (Bass et al., 2006). These findings, as well as those of Tripathy et al. (2010) in which participatory women's groups had a strong impact on maternal psychological distress at a population level, suggest that group-based interventions can also be effective in treating common mental disorders.

Scaling up interventions with groups or with lay or community health workers could therefore provide new opportunities to treat common mental disorders, including postpartum depression, in low and middle-income countries. Screening for postpartum depression using simple tools such as the SRQ20 or GHQ12, which have been shown to reliably identify depression cases when compared to the Revised Clinical Interview Schedule (Patel et al., 2008), could also be integrated with maternal health care, especially for mothers at high risk, e.g. those who have experienced the loss of an infant, or one or more of the risk factors identified in this study, and these mothers referred to appropriate community or primary care interventions.

This study had two main limitations: we did not collect data on a number of factors known to influence the risk of postpartum psychological distress, such as antepartum depression, domestic violence or a mother's past birth history, including the sex of her previous children, and were therefore unable to quantify their importance as predictors of postpartum distress. The fact that we did not collect data on violence is of particular concern, since studies indicate that the prevalence of exposure to physical violence among Scheduled Tribe women may be as high as 25%, and recent intimate partner violence was strongly associated with common mental disorders in a large Indian population-based study (Babu and Kar, 2009, NFHS-3, 2005–6, Shidaye and Patel, 2010). Finally, the cross-sectional and observational nature of the study means that we are only able to report associations between predictors and psychological distress and cannot make claims of causality.

Rural tribal mothers who experience an infant death, social disadvantage or health problems during the perinatal period appear to be at increased risk of psychological distress and require access to quality reproductive health services with integrated mental health care. Further research should focus on testing strategies to integrate mental health interventions into primary care and reproductive health services in underserved areas of India.

## Role of funding source

Funding for the study from which our data are drawn was provided by the Health Foundation (UK), the Wellcome Trust, the UK Department for International Development and the Big Lottery Fund (UK). The funders had no role in the design of the study, data collection, data analysis, interpretation, or writing up of the findings. The corresponding author had access to all the data and had final responsibility for the decision to submit for publication.

## Conflict of interest

All authors declare that they have no conflicts of interest.

## Figures and Tables

**Table 1:** Socio-demographic characteristics of mothers included in the study (N = 5801).

Characteristics	Total (N = 5801)
Age in years, mean (SD)	25.5 (5.3)
Parity, mean (SD)	2.9 (1.96)
Education, n (%)	
None	3848 (66.3)
Primary	304 (5.2)
Secondary	1451 (25.0)
Higher secondary or higher	198 (3.4)
Can read, n (%)	
No	3952 (68.1)
Yes	1849 (31.9)
Caste/tribal status, n (%)	
Scheduled Tribe	3932 (67.8)
Scheduled Caste	258 (4.4)
Other Backward Class	1591 (27.2)
Other	20 (0.3)
Owns land, n (%)	
Yes	5099 (87.9)
No	702 (12.1)
Household assets, n (%)	
Bicycle	4491 (79.1)
Radio	1768 (30.5)
Electricity	876 (15.1)
Motorcycle	500 (8.6)
Below poverty line card, n (%)	
Yes	2492 (42.0)
No	3309 (57.4)
Main decision-maker(s) for daily household expenditure	
Mother	308 (5.3)
Mother and husband (jointly)	2137 (36.8)
Husband and in-laws	3259 (56.2)
Main decision-maker(s) for care-seeking in case of maternal illness	
Mother	119 (2.0)
Mother and husband (jointly)	1923 (33.1)
Husband and in-laws	3680 (63.4)

**Table 2:** Univariable and multivariable analyses of association of factors with psychological distress — all mothers (N = 5801).

Variable	Prevalence n (%)	Presence of distress (K10 > 15) n (%)	Unadjusted OR with 95%CI — univariable analysis	p-value	Adjusted OR with 95%CI — multivariable analysis	p-value
*Socio-economic factors*						
Maternal age in years^a^						
15–22	1955 (33.7)	227 (11.6)	1	0.113^b^	1	0.040^b^
23–27	1839 (31.7)	219 (11.9)	1.12 (0.92–1.39)		1.27 (1.02–1.59)	
28–32	1274 (22.0)	141 (11.1)	1.04 (0.83–1.32)		1.18 (0.91–1.53)	
33–39	575 (9.9)	72 (12.5)	1.37 (1.01–1.85)		1.44 (1.03–2.07)	
Maternal education^a^						
None	3848 (66.3)	427 (11.1)	1	0.055^b^		
Primary	304 (5.2)	54 (17.8)	1.34 (0.96–1.98)			
Secondary +	1649 (28.4)	188 (11.4)	0.87 (0.54–1.41)			
Literacy						
Can read	1849 (31.9)		1			
Cannot read	3952 (68.1)		1.20 (0.76–1.89)	0.202		
Caste or tribal group (ST: Scheduled Tribe; SC: Scheduled Caste; OBC: Other Backward Class)						
ST	3932 (67.8)	430 (10.9)	1	0.318^b^		
SC	258 (4.4)	44 (17.0)	1.32 (0.92–1.91)			
OBC	1581 (27.2)	191 (12.1)	1.10 (0.90–1.35)			
Other	30 (0.5)	4 (13.3)	0.91 (0.30–2.75)			
Below the Poverty Line card ownership						
No	3309 (57.0)	303 (12.2)	1			
Yes	2492 (43.0)	366 (11.1)	1.01 (0.85–1.21)	0.847		
Land ownership^a^						
Yes	5099 (87.9)	578 (11.3)	1			
No	702 (12.1)	91 (13.0)	1.19 (0.92–1.54)	0.184		
Asset ownership (fourth asset quartile: poorest)^a^						
First	1396 (24.1)	155 (11.1)	1	0.017^b^	1	0.019^b^
Second	1278 (22.0)	166 (13.0)	1.15 (1.14–1.89)		1.56 (1.19–2.05)	
Third	1085 (18.7)	99 (9.1)	1.07 (0.80–1.44)		1.17 (0.85–1.61)	
Fourth	2042 (35.2)	249 (12.2)	1.46 (1.14–1.87)		1.52 (1.16–1.99)	

*Gender-related factors*						
Main decision-makers for daily household expenditures						
Mother	308 (5.3)	47 (15.3)	1	0.002^b^		
Mother and husband jointly	2137 (36.8)	213 (10.0)	0.66 (0.44–0.99)			
Husband and in-laws	3259 (56.2)	400 (12.3)	0.55 (0.37–0.80)			
Main decision-makers for care-seeking in case of maternal illness						
Mother	119 (2.0)	26 (21.8)	1	0.455^b^		
Mother and husband jointly	1923 (33.1)	202 (10.5)	0.29 (0.17–0.47)			
Husband and in-laws	3680 (63.4)	431 (11.7)	0.37 (0.23–0.59)			
Food restriction in the postpartum period (one meal a day)						
No	4079 (70.3)	496 (12.2)	1			
Yes	1722 (29.7)	173 (10.0)	0.90 (0.72–1.12)	0.356		

*Physical and reproductive health factors*						
Parity^a^						
1–2 children	3023 (52.1)	362 (12.0)	1	0.507^b^		
3–4 children	1683 (29.0)	185 (11.0)	1.00 (0.82–1.22)			
4 + children	1095 (18.9)	122 (11.1)	1.09 (0.87–1.38)			
Health problem during pregnancy^a^^,^^c^						
No	3116 (53.7)	246 (7.9)	1		1	
Yes	2685 (46.3)	423 (15.7)	2.29 (1.91–2.76)	< 0.005	1.98 (1.62–2.41)	< 0.005
Health problem during delivery^a^^,^^d^						
No	3767 (64.9)	326 (8.6)	1		1	
Yes	2034 (35.1)	343 (16.9)	2.03 (1.69–2.43)	< 0.005	1.63 (1.34–1.99)	< 0.005
Health problem in the postpartum period^a^^,^^e^						
No	4030 (69.5)	408 (10.1)	1		1	
Yes	1771 (30.5)	261 (14.7)	1.88 (1.56–2.27)	< 0.005	1.50 (1.22–1.83)	< 0.005
Caesarean section						
No	5702 (98.3)	646 (11.3)	1		1	
Yes	99 (1.7)	23 (23.2)	2.53 (1.52–4.21)	< 0.005	1.95 (1.12–3.40)	0.018
Consumption of local alcohol (*handia*) in pregnancy^a^						
No	3253 (56.0)	383 (11.8)	1			
Yes	2548 (43.9)	286 (11.2)	1.07 (0.89–1.29)	0.449		
Mother wanted the pregnancy^a^						
Yes	5147 (88.7)	572 (11.1)	1		1	
No	654 (11.3)	97 (14.8)	1.42 (1.10–1.82)	0.006	1.49 (1.12–1.97)	0.005
Father wanted the pregnancy^a^						
Yes	5178 (89.3)	580 (11.2)	1			
No	623 (10.7)	89 (14.3)	1.36 (1.05–1.77)	0.019		

Gender of infant						
Male	2974 (51.3)	351 (11.8)	1			
Female	2827 (48.7)	318 (11.2)	0.95 (0.80–1.13)	0.571		

*Factors related to birth outcome*						
Infant perceived to be smaller than average						
No	5436 (93.7)	578 (10.6)	1		1	
Yes	365 (6.3)	91 (24.9)	2.74 (2.08–3.62)	< 0.005	1.42 (1.04–1.94)	0.026
Perinatal loss (stillbirth or neonatal death)						
No	5345 (92.1)	490 (9.2)	1		1	
Yes	456 (7.9)	179 (39.2)	8.37 (6.64–10.5)	< 0.005	7.06 (5.51–9.04)	< 0.005

a Interaction term with infant loss (stillbirth or neonatal death) significant at p < 0.1.

b Overall p-value for categorical variable.

c Health problems during pregnancy include: severe pain in stomach; excessive vomiting; fever for more than 24 h; vaginal bleeding; jaundice; reduced/no foetal movement; and self-reported symptoms of malaria.

d Health problems during delivery include: high fever in the 3 days before labour, foul smelling vaginal discharge; prolonged labour; fits/convulsions; vaginal bleeding; retained placenta; tear around birth passage; and umbilical cord around baby's neck.

e Health problems in the postpartum period include: severe pain in stomach; fever for more than 24 h; foul smelling discharge; vaginal bleeding; leaking from vagina.

**Table 3:** Univariable and multivariable analyses of association of factors with psychological distress, stratified by presence or absence of infant loss.

Mothers with living infants (n = 5345)							Mothers with an infant loss (n = 456)					
Variable	Prevalence n (%)	Presence of distress (K10 > 15), n (%)	Unadjusted OR with 95%CI — univariable analysis	p-value	Adjusted OR with 95%CI — multivariable analysis	p-value	Prevalence n (%)	Presence of distress (K10 > 15), n (%)	Unadjusted OR with 95%CI — univariable analysis	p-value	Adjusted OR with 95%CI — multivariable analysis	p-value
*Socio-economic factors*												
Maternal age in years												
15–22	1771 (33.1)	146 (8.2)	1	0.001^a^	1	0.004^a^	184 (40.3)	81 (44.0)	1	0.125^a^	1	0.151^a^
23–27	1710 (32.0)	166 (9.7)	1.34 (1.05–1.72)		1.37 (1.07–1.77)		129 (28.3)	53 (41.1)	1.04 (0.62–1.73)		1.03 (0.62–1.72)	
28–32	1184 (22.1)	109 (9.2)	1.25 (0.95–1.65)		1.24 (0.93–1.67)		90 (19.7)	32 (35.6)	0.95 (0.58–1.58)		1.03 (0.57–1.85)	
33–39	530 (9.9)	60 (11.3)	2.00 (1.41–2.81)		1.82 (1.26–2.64)		45 (9.9)	12 (26.7)	0.44 (0.20–0.97)		0.43 (0.20–0.96)	
Missing	150 (2.8)	9 (6.0)	–		–		8 (1.7)	1 (12.5)	–	–		
Maternal education												
None	3535 (66.1)	305 (8.6)	1	0.120			313 (68.6)	122 (38.9)	1	0.763^a^		
Primary	272 (5.1)	44 (16.2)	1.51 (1.03–2.21)				32 (7.0)	10 (31.2)	0.65 (0.27–1.54)			
Secondary +	1538 (28.8)	141 (9.2)	0.80 (0.63–1.03)				111 (24.3)	47 (42.3)	0.58 (0.65–1.58)			
Literacy												
Can read	1713 (32.0)	169 (9.9)	1				136 (29.8)	56 (41.2)	1			
Cannot read	3632 (67.9)	321 (8.8)	1.13 (0.90–1.42)	0.290			320 (70.2)	123 (38.4)	1.10 (0.68–1.75)	0.701		
Caste or tribal group (ST: Scheduled Tribe; SC: Scheduled Caste; OBC: Other Backward Class)												
ST	3636 (68.0)	322 (8.9)	1	0.888^a^			296 (64.9)	108 (36.5)	1	0.090^a^		
SC	230 (4.3)	31 (13.5)	1.17 (0.76–1.79)				28 (6.1)	13 (46.4)	1.61 (0.68–3.78)			
OBC	1451 (27.1)	134 (9.2)	1.01 (0.80–1.29)				130 (28.5)	57 (43.8)	1.47 (0.90–2.39)			
Other	28 (0.5)	3 (10.7)	0.82 (0.24–2.88)				2 (0.4)	1 (50.0)	2.49 (0.13–47.0)			
Below the Poverty Line card ownership												
No	3042 (56.9)	258 (8.5)	1				189 (41.4)	71 (37.6)	1			
Yes	2303 (43.1)	232 (10.1)	0.94 (0.77–1.16)	0.598			267 (58.5)	108 (40.5)	1.24 (0.80–1.92)	0.338		
Land ownership												
Yes	4703 (88.0)	418 (8.9)	1				396 (86.8)	160 (40.4)	1			
No	642 (12.0)	72 (11.2)	1.29 (0.96–1.73)	0.081			60 (13.2)	19 (31.7)	0.85 (0.44–1.65)	0.638		
Asset quartiles (fourth quartile: poorest)												
First	1305 (24.4)	114 (8.7)	1	0.015^a^	1	0.009^a^	91 (20.0)	41 (45.0)	1	0.347^a^		
Second	1168 (21.8)	123 (10.5)	1.54 (1.15–2.07)		1.75 (1.28–2.38)		110 (24.1)	43 (39.1)	0.97 (0.52–1.83)			
Third	1005 (18.8)	72 (7.2)	1.11 (0.79–1.56)		1.26 (0.88–1.81)		80 (17.5)	27 (33.7)	0.68 (0.34–1.38)			
Fourth	1867 (34.9)	181 (9.7)	1.56 (1.17–2.07)		1.69 (1.25–2.30)		175 (38.4)	68 (38.9)	0.79 (0.44–1.44)			

*Gender-related factors*												
Main decision-makers for daily household expenditure												
Mother	282 (5.3)	38 (13.5)	1	< 0.005^a^			26 (5.7)	9 (34.6)	1	0.873^a^		
Mother and husband jointly	1977 (37.0)	151 (7.6)	0.54 (0.34–0.85)				160 (35.1)	62 (38.7)	1.61 (0.54–4.76)			
Husband and in-laws	2997 (56.1)	295 (9.8)	0.43 (0.28–0.66)				262 (57.5)	105 (40.1)	1.41 (0.51–3.89)			
Main decision-makers for healthcare seeking in case of maternal illness												
Mother	112 (2.1)	24 (21.4)	1	0.079^a^			7 (1.5)	2 (28.6)	1	0.253^a^		
Mother and husband jointly	1777 (33.2)	144 (8.1)	0.21 (0.12–0.37)				146 (32.0)	58 (39.7)	1.33 (0.22–8.00)			
Husband and in-laws	3384 (63.3)	315 (9.3)	0.27 (0.16–0.45)				296 (64.9)	116 (39.2)	1.77 (0.31–10.1)			
Food restriction in the postpartum period (one meal a day)												
No	3744 (70.0)	363 (9.7)	1				335 (73.5)	133 (39.7)	1			
Yes	1601 (29.9)	127 (7.9)	0.89 (0.69–1.15)	0.359			121 (26.5)	46 (38.0)	1.14 (0.68–1.91)	0.622		

*Physical and reproductive health factors*												
Parity												
1–2 children	2762 (51.7)	250 (9.0)	1	0.013^a^			261 (57.2)	112 (42.9)	1	0.045^a^		
3–4 children	1570 (29.4)	139 (8.8)	1.11 (0.88–1.40)				113 (24.8)	46 (40.7)	0.97 (0.59–1.58)			
4 + children	1013 (18.9)	101 (10.0)	1.41 (1.08–1.84)				82 (18.0)	21 (25.6)	0.50 (0.27–0.91)			
Health problem during pregnancy^b^												
No	2921 (54.6)	174 (6.0)	1		1		195 (42.8)	72 (36.9)	1			
Yes	2424 (45.3)	316 (13.0)	2.56 (2.06–3.19)	< 0.005	2.35 (1.88–2.94)	< 0.005	261 (57.2)	107 (41.0)	1.14 (0.73–1.78)	0.563		
Health problem during delivery^c^												
No	3535 (66.1)	245 (6.9)	1		1		232 (50.9)	81 (34.9)	1			
Yes	1810 (33.9)	245 (13.5)	1.84 (1.49–2.28)	< 0.005	1.71 (1.37–2.13)	< 0.005	224 (49.1)	98 (43.7)	1.59 (1.03–2.45)	0.036	1.56 (1.01–2.42)	0.046
Health problem in the postpartum period^d^												
No	3760 (70.3)	304 (8.1)	1		1		270 (59.2)	104 (38.5)	1			
Yes	1585 (29.6)	186 (11.7)	1.91 (1.54–2.38)	< 0.005	1.71 (1.36–2.16)	< 0.005	186 (40.8)	75 (40.3)	1.23 (0.79–1.91)	0.358		
Caesarean section												
No	5260 (98.4)	473 (8.9)	1		1		442 (96.9)	173 (39.1)	1			
Yes	85 (1.6)	17 (20.0)	2.79 (1.54–5.07)	0.001	2.51 (1.35–4.65)	0.004	14 (3.1)	6 (42.9)	1.43 (0.43–4.71)	0.554		
Consumption of local alcohol (*handia*) in pregnancy												
No	2990 (55.9)	274 (9.2)	1				263 (57.7)	109 (41.4)	1			
Yes	2355 (44.1)	216 (9.2)	1.16 (0.94–1.45)	0.159			193 (42.3)	70 (36.3)	0.81 (0.51–1.29)	0.384		
Mother wanted the pregnancy												
Yes	4739 (88.7)	404 (8.5)	1		1		408 (89.5)	168 (41.2)	1			
No	606 (11.3)	86 (14.2)	1.96 (1.48–2.58)	< 0.005	1.83 (1.36–2.47)	< 0.005	48 (10.5)	11 (22.9)	0.32 (0.14–0.70)	0.004		
Father wanted the pregnancy												
Yes	4768 (89.2)	412 (8.6)	1				410 (89.9)	168 (41.0)	1			
No	577 (10.8)	78 (13.5)	1.82 (1.37–2.43)	< 0.005			46 (10.1)	11 (23.9)	0.41 (0.19–0.88)	0.023		

Gender of infant												
Male	2711 (50.7)	245 (9.0)	1				263 (57.7)	106 (40.3)	1			
Female	2634 (49.3)	245 (9.3)	1.04 (0.86–1.27)	0.646			193 (42.3)	73 (37.8)	0.92 (0.61–1.40)	0.708		

*Factors related to birth outcome*												
Infant perceived to be smaller than average												
No	5089 (95.2)	447 (8.8)	1		1		347 (76.1)	131 (37.7)	1			
Yes	256 (4.8)	43 (16.8)	1.81 (1.24–2.65)	0.002	1.50 (1.01–2.23)	0.043	109 (23.9)	48 (44.0)	1.28 (0.78–2.08)	0.319		

a Overall p-value for categorical variable.

b Health problems during pregnancy include: severe pain in stomach; excessive vomiting; fever for more than 24 h; vaginal bleeding; jaundice; reduced/no foetal movement; and self-reported symptoms of malaria.

c Health problems during delivery include: high fever in the 3 days before labour, foul smelling vaginal discharge; prolonged labour; fits/convulsions; vaginal bleeding; retained placenta; tear around birth passage; and umbilical cord around baby's neck.

d Health problems in the postpartum period include: severe pain in stomach; fever for more than 24 h; foul smelling discharge; vaginal bleeding; leaking from vagina.
